# Genome sequence of white spot syndrome virus (WSSV) infecting cultured black tiger shrimp (*Penaeus monodon*) in Bangladesh

**DOI:** 10.1128/mra.01211-23

**Published:** 2024-03-19

**Authors:** S. M. Rafiqul Islam, Robel Ahmed, Farjana Sharmen, Md. Mobarok Hossain, Kallyan Chakma, Afroza Akter Tanni, Md. Ashikur Alim Akash, Mohammad Enayet Hossain, M. Shah Nawaz Chowdhury, AMAM Zonaed Siddiki, Anwar Hossain, Shankar C. Mandal, Keith A. Crandall, Ali Rahnavard, SM Sharifuzzaman, Adnan Mannan

**Affiliations:** 1 Department of Genetic Engineering and Biotechnology, University of Chittagong, Chattogram, Bangladesh; 2 Next-generation Sequencing, Research and Innovation Laboratory Chittagong (NRICh), Biotechnology Research and Innovation Centre (BRIC), University of Chittagong, Chattogram, Bangladesh; 3 International Centre for Diarrhoeal Disease Research, Bangladesh (ICDDR,B), Dhaka, Bangladesh; 4 Institute of Marine Sciences, University of Chittagong, Chattogram, Bangladesh; 5 Department of Pathology and Parasitology, Genomics Research Group, Chattogram Veterinary and Animal Sciences University, Chattogram, Bangladesh; 6 Department of Fisheries, Aquaculture Genomics Laboratory, University of Dhaka, Dhaka, Bangladesh; 7 Department of Biostatistics and Bioinformatics, Computational Biology Institute, Milken Institute School of Public Health, George Washington University, Washington, District of Columbia, USA; DOE Joint Genome Institute, Berkeley, California, USA

**Keywords:** white spot syndrome virus, shrimp, Bangladesh

## Abstract

The white spot syndrome virus (WSSV) is a causative agent of white spot disease (WSD) in crustaceans, especially in cultivated black tiger shrimp (*Penaeus monodon*), leading to significant economic losses in the aquaculture sector. The present study describes four whole genome sequences of WSSV obtained from coastal regions of Bangladesh.

## ANNOUNCEMENT

White spot syndrome virus (WSSV) is a rod-shaped, double-stranded circular DNA virus. It is the only known member of the genus *Whispovirus* belonging to the family *Nimaviridae*. In Bangladesh, WSSV was identified in 1994 at a semi-intensive shrimp farms in the southern coastal region at Cox’s Bazar, and then, it spread to the southwest coastal areas (1). A study conducted on the circulatory WSSV highlights the concerning extent of its infection in Bangladeshi cultivated shrimps, exceeding critical thresholds and resulting in ~20% drop in national shrimp production (2).

In this study, WSSV-suspected shrimp samples were collected from two shrimp farms located in the coastal regions of Satkhira (hereafter SAT) in July 2021 and Cox’s Bazar (hereafter COX) in June 2023. The presence of WSSV was initially determined by observing the phenotypic symptoms (white spots on shrimp shells) of white spot disease (WSD) and then confirmed with the PCR technique by targeting the VP28 gene (2, 3). Genomic DNA (gDNA) was extracted from abdominal tissue from individual shrimp (two shrimps per farm for both farms resulting in four total samples) using the Qiagen Tissue DNA Purification Kit (Qiagen QIAamp, Germany). To ensure the samples were WSSV positive, the extracted gDNA was amplified by conventional PCR with the primer pairs: VP28-FW (5′-TGTGACCAAGACCATCGAAAC-3′) and VP28-RV (5′-TCGGTCTCAGTGCCAGAGTA-3′), targeting gene VP28 (3). For whole genome sequencing, DNA libraries were prepared by using the tagmentation and PCR-based Illumina DNA Prep Kit (Illumina, San Diego, CA, USA), and sequencing was performed on the Illumina NovaSeq 6000 platform generating 2 × 150 bp paired-end reads.

Unless otherwise specified, all tools used in sequencing data analysis were run with default parameters. Initially, low-quality sequence reads and adapter sequences were removed using the Trimmomatic v 0.40 tool (4). The quality of trimmed reads was checked using FastQC v 0.11.9 tool (5). The viral reads were initially detected from the sequencing reads by Kraken2 (6) using the viral database, and then, the viral reads were extracted by mapping the reads to the WSSV reference genome (NC_003225.3) (7). *De novo* assembly was performed using the SPAdes v 3.15.5 tool (8), and QUAST v 5.0.2 tool (9) was used to determine the statistics of assembled genomes. A consensus sequence was generated by aligning contiguous sequences to the WSSV reference sequence (NC_003225.3). Contigs representing WSSV were identified by comparing assembled sequences to a reference WSSV genome NC_003225.3 using ABACAS (10). After aligning the contigs with the reference sequence (NC_003225.3), 95%–98% of genome coverage was found and the remaining portions of the sequences were derived from the reference sequence. The coverage depth for each genome was calculated from the mapped file generated by Bowtie2 v 2.3.5.1 (11) using SAMtools v 1.10 (12). The sequence statistics including total length, read depth, genome coverage (X), GC content (%), SNPs, and accession numbers for each WSSV genome are summarized in Table 1.

**TABLE 1:** Summary statistics and GenBank accession numbers of WSSV genomes^*a*^

WSSV strain	Origin of sample	Sample ID	Reference length (bp)	Total length (bp)	Total reads	Mapped reads	Genome coverage (X)	GC content (%)	SNPs	GenBank accession no.
WSSV_BD_CU_SAT-2	Satkhira, Bangladesh	BD_SAT_3	309,286	309,286	1,559,766	140,432	66	41.1	221	PP134842
WSSV_BD_CU_SAT-1	Satkhira, Bangladesh	BD_SAT_1	309,286	309,286	1,748,558	69,547	32.7	41.1	215	PP134841
WSSV_BD_CU_COX-2	Cox’s Bazar, Bangladesh	BD_COX_4	309,286	309,286	944,176	188,102	88.1	41.1	228	PP134839
WSSV_BD_CU_COX-1	Cox’s Bazar, Bangladesh	BD_COX_2	309,286	309,286	1,198,384	326,314	100	41.1	230	PP134840

^*a*^ COX, Cox’s Bazar; SAT, Satkhira; WSSV, white spot syndrome virus; SNPs, single nucleotide polymorphisms.

For the phylogenetic analysis, sequence reads from a total of 35 WSSV strains were downloaded from the NCBI Sequence Read Archive (SRA) database, including five Bangladeshi samples (four from this study). Consensus genomes were estimated for each strain as described above. Phylogenetic relationships among the WSSV genomes were estimated using the maximum likelihood method (13, 14) implemented in RAxML (15). Phylogenetic estimate used the GTR+Γ substitution model of evolution (16), and node support was assessed utilizing the bootstrap approach (17) at RAxML with 100 pseudo-replications. Visualization of the tree was accomplished using iTOL (18).

**Fig 1:**
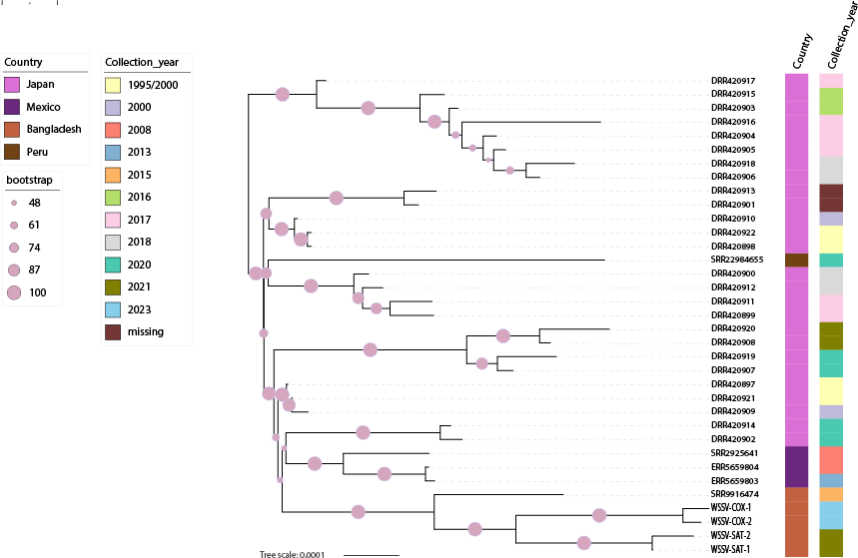
Maximum likelihood tree showing relationships among WSSV genomes. The colored squares indicate the isolation source and collection date separately. Maximum likelihood bootstrap values are shown as purple circles. The sequenced samples for this study are denoted as COX-1, COX-2, SAT-1, and SAT-2.

The phylogenetic placement of these four new WSSV samples (Fig. 1) relative to other WSSV genomes shows these new strains form a phylogenetically distinct cluster. Indeed, across the phylogeny, WSSV strains segregate by country, creating sub-clusters that further represent their diversity over time.

## Data Availability

This whole genome sequencing project was submitted to NCBI/GenBank under BioProject accession number PRJNA1029923. The raw reads of genome sequences for WSSV_BD_CU_SAT2, WSSV_BD_CU_SAT1, WSSV_BD_CU_COX2, and WSSV_BD_CU_COX1 were deposited in the SRA under accession numbers SRR26436527, SRR26436528, SRR26436529, and SRR26436530, respectively. Assembled genomes are available in GenBank under accession numbers PP134842, PP134841, PP134839, and PP134840.
